# Magnetic dynamics of ferromagnetic long range order in icosahedral quasicrystal

**DOI:** 10.1038/s41598-022-14796-5

**Published:** 2022-06-24

**Authors:** Shinji Watanabe

**Affiliations:** Department of Basic Sciences, Kyushu Institute of Technology, Kitakyushu, Fukuoka, 804-8550 Japan

**Keywords:** Physics, Condensed-matter physics, Magnetic properties and materials

## Abstract

Quasicrystals lack translational symmetry and have unique lattice structures with rotational symmetry forbidden in periodic crystals. The electric state and physical property are far from complete understanding, which are the frontiers of modern matter physics. Recent discovery of the ferromagnetic long-range order in the rare-earth based icosahedral quasicrystal has made the breakthrough. Here, we first reveal the dynamical as well as static magnetic structure in the ferromagnetic long-range order in the terbium-based quasicrystal. The dynamical structure factor exhibits highly structured energy and wavenumber dependences beyond the crystalline-electric-field excitation. We find the presence of the magnetic excitation mode analog to magnon with unique hierarchical structure as well as the localized magnetic excitation with high degeneracy in the quasicrystal. Non-collinear and non-coplanar magnetic structure on the icosahedron is discovered to give rise to non-reciprocal magnetic excitation in the quasicrystal as well as non-reciprocal magnon in the periodic cubic 1/1 approximant. These findings afford illuminating insight into the magnetic dynamics in the broad range of the rare-earth-based quasicrystals and approximants.

The quasicrystal (QC) discovered in 1984^1^ has a unique lattice structure with rotational symmetry forbidden in periodic crystals. Although understanding of the atomic configurations has proceeded^2,3^, their electronic states and physical properties remain elusive, which provide challenging and fascinating frontier of modern matter physics. This is because the Bloch theorem based on translational symmetry in periodic crystals can no longer be applied. One of the remaining significant issues has been whether the magnetic long-range order is realized in the QC^4^.

Interestingly, periodic crystals with the local atomic configuration common to the QC is known to exist, which is called the approximant crystal (AC). In the rare-earth based ACs, the magnetic long-range order has been observed in Cd$_6$R (R = Pr, Nd, Sm, Gd, Tb, Dy, Ho, Eu, and Tm)^5–8^ and Au–SM–R (SM = Si, Al, Ge, and Sn; R = Gd, Tb, Dy, and Ho)^9–12^. The 4f electrons at the rare-earth R site are responsible for the magnetism.

Recently, ferromagnetic (FM) long-range order has been discovered experimentally in the QC Au–Ga–R (R = Tb, Gd)^13^. The temperature dependence of the magnetic susceptibility shows the positive Curie-Weiss temperature, which indicates the FM interaction working between the magnetic moments of the 4f electrons.

Theoretically, so far the spin model and the Hubbard model in low-dimensional systems such as the Fibonacci chain and the Penrose lattice or small clusters have been extensively studied^14–29^. However, the magnetism on the real rare-earth based three dimensional QCs remains unresolved because the lack of the microscopic theory of the crystalline electric field (CEF), which is essentially important for the 4f electronic state, has prevented us from understanding the magnetic property. Recently, the theory of the CEF in the rare-earth based QC and AC has been developed on the basis of the point charge model^30^. This has succeeded in formulating the full CEF Hamiltonian of any rare-earth ion in terms of the total angular momentum, which enables us to analyze the CEF accurately. By applying this formulation to the QC Au–SM–Tb and the AC, the CEF has been analyzed theoretically^31,32^. Then, it has been revealed that the magnetic anisotropy arising from the CEF plays a key role in realizing unique magnetic states on the icosahedron (IC) at whose vertices the Tb atoms are located. By analyzing the magnetic model considering the magnetic anisotropy in the QC Au–SM–Tb, the FM long-range order of the ferrimagnetic state has been discovered theoretically, as shown in Fig. 1a^31^. The magnetic moments on the IC are aligned as Fig. 1b, where the non-collinear and non-coplanar ferrimagnetic state is realized with the total magnetization per the IC being finite along the (111) direction. This magnetic structure has actually been identified in the 1/1 AC Au$_{70}$Si$_{17}$Tb$_{13}$ by the neutron measurement, where the magnetic moment at each Tb site is lying in the mirror plane and is $80^{\circ }$ tilted from the pseudo 5-fold axis (see Fig. 1b) forming the ferrimagnetic state on the IC^12^. This state is uniformly distributed at the center and the corner in the unit cell of the body-center-cubic (bcc) lattice, which forms the FM-long range order in the 1/1 AC, as shown in Fig. 1c^12^.

**Figure 1:**
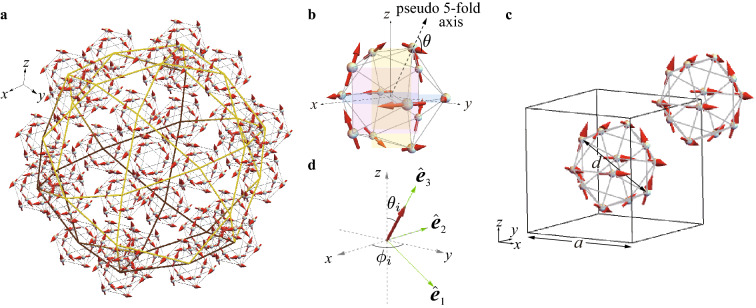
(**a**) FM long-range order of the ferrimagnetic state in the QC. The magnetic moments at the 12 vertices of the IC located at the vertices of the icosidodecahedron and also at the center. Green (brown) lines connect the vertices of the icosidodecahedron in the front (back) side. (**b**) The ferrimagnetic state in the IC. The vector passing through the Tb site from the center of the IC is the pseudo 5-fold axis drawn as the dashed line with an arrow, which is in the mirror plane colored by yellow surface. The same is applied to each Tb site where the mirror planes are colored by pink and light blue surfaces. The direction of the magnetic moment is defined as the angle $\theta$ from the pseudo 5-fold axis. The $\theta =80^{\circ }$ case is illustrated. (**c**) In the 1/1 AC, the ferrimagnetic state for $\theta =80^{\circ }$ on the IC is located at the center and corner at the bcc unit cell with *a* being the lattice constant $a=14.726~$Å. (**d**) Local coordinate introduced at each Tb site where $\hat{{\varvec{e}}}_3$ is set to be the unit vector along the direction of the magnetic moment and $\hat{{\varvec{e}}}_1$ and $\hat{{\varvec{e}}}_2$ are orthogonal unit vectors each other (see “Methods” section). The polar angle at the *i*th Tb site is defined as $(\theta _i,\phi _i)$ in the global *xyz* coordinate. (This figure is created by using Adobe Illustrator CS5 Version 15.1.0.).

Although the arrangement of the magnetic moments of the FM order has been identified in the real space as Fig. 1a, the magnetic structure in the reciprocal space has not been elucidated^31^. Furthermore, the dynamical magnetic structure factor has not been clarified theoretically nor experimentally. In periodic crystals, the excitation in the magnetically ordered state is known as “magnon”. In the QC, the wavenumber is no longer a good quantum number and hence it is interesting whether the magnon-like excitation exists in the reciprocal space. It is also curious whether the magnetic excitation unique to the icosahedral QC exists.

So far, the lattice dynamics in the QC has been studied by inelastic X-ray- and neutron-scattering experiments^33,34^. Theoretical studies of the lattice dynamics have also been reported^33,35^. As for the magnetic dynamics, the dynamical structure factor has been calculated in the ferromagnetically aligned spins on the Fibonacci chain^36^ and antiferromagnetically aligned spins on the two-dimensional octagonal tiling^37^.

However, little has been known about the magnetic dynamics in the real three-dimensional QC experimentally nor theoretically.

In this article, we *for the first time* clarify the magnetic dynamics of the FM long-range order in the three-dimensional icosahedral QC. By analyzing the magnetic model taking into account the effect of the magnetic anisotropy arising from the CEF in the QC Au-SM-Tb, we clarify the dynamical as well as static magnetic structure. The dynamical structure factor is shown to exhibit highly structured energy and wavenumber dependences. We find that the pseudo magnon mode as well as the localized magnetic-excitation mode exists in the QC. We also find non-reciprocal magnetic excitation in the QC as well as the non-reciprocal magnon in the 1/1 AC. These findings provide insight into the understanding of the recently discovered FM long-range order in the QC Au$_{65}$Ga$_{20}$Tb$_{15}$^13^.

## Results

### Lattice structure of quasicrystal

Let us start with the lattice structure of the QC. Although the detailed lattice structure of the QC Au$_{65}$Ga$_{20}$Tb$_{15}$ has not been solved experimentally, the Tb lattice is considered to form the Cd$_{5.7}$Yb-type QC^3^ . Figure 1a shows the main structure of the QC where the Tb-12 cluster i.e., the IC is located at 30 vertices of the icosidodecahedron. In the Cd$_{5.7}$Yb-type QC, there exist a few other ICs as well as Tb sites located between ICs. In this study, as a first step of analysis, we consider the Tb sites on ICs shown in Fig. 1a with the total lattice number is $N=12\times 30=360$. In this study, we employ the real Tb configuration of the IC determined in the 1/1 AC Au$_{70}$Si$_{17}$Tb$_{13}$^12^ and locate them at the 30 vertices of the $\tau ^3$-times enlarged icosidodecahedron in the Tsai-type cluster of Au$_{70}$Si$_{17}$Tb$_{13}$. Here, $\tau =(1+\sqrt{5})/2$ is the golden mean.

### Minimal model of rare-earth based icosahedral quasicrystal

Then, we consider the minimal model for the magnetism of the Tb-based QC as

1 $$\begin{aligned} H=\sum _{\langle i,j\rangle }J_{ij}{{\varvec{S}}}_i\cdot {{\varvec{S}}_j}-D\sum _{i}({{\varvec{S}}}_i\cdot \hat{{\varvec{e}}}_3)^2, \end{aligned}$$

where $J_{ij}$ is the exchange interaction between the “spin” ${{\varvec{S}}}_i$ on the *i*th Tb site and ${{\varvec{S}}}_j$ on the *j*th Tb site. Here ${{\varvec{S}}}_i$ expresses the total angular momentum ${\varvec{J}}$. Since the Hund’s rule tells us that the ground multiplet of the Tb$^{3+}$ ion with $4f^8$ configuration is given by $J=6$, we set $S=6$. We consider the nearest neighbor (N.N.) interaction $J_1$ and the next N.N. (N.N.N.) interaction $J_2$ not only for the intra IC but also for the inter IC as discussed in Ref.^31^. The second term in Eq. (1) represents the magnetic anisotropy arising from the CEF, where ${{\varvec{e}}}_3$ is the unit vector set to be along the direction of the ordered moment at each Tb site (see Fig. 1d) and *D* is the parameter of anisotropy.

In the strong anisotropy limit of the model (1), it has been confirmed that the FM long-range order of the ferrimagnetic state shown in Fig. 1a is stabilized in the wide parameter region of $J_2/J_1$ for $64.1^{\circ }\le \theta \le 80^{\circ }$ of the ground-state phase diagram (see Fig. S3a in Supplementary Information)^31^. In this study, we analyze the FM ground state of the ferrimagnetic state with $\theta =80^{\circ }$ (see Fig. 1b) in the QC lattice shown in Fig. 1a under the open boundary condition.

### Magnetic excitation in quasicrystal

Since the ferrimagnetic state is the non-collinear alignment of “spins”, it is convenient to introduce the local coordinate at each Tb site where the ${\hat{e}}_3$ axis is taken as the ordered “spin” direction as shown in Fig. 1d (see “Methods” section). Then, by applying the Holstein-Primakoff transformation^38^ to *H*, the “spin” operators are transformed to the boson operators as $S_i^{+}=\sqrt{2S-n_i}a_i$, $S_i^{-}=a_i^{\dagger }\sqrt{2S-n_i}$ and ${{\varvec{S}}}_i\cdot \hat{{\varvec{e}}}_3^i=S-n_i$ with $n_i\equiv a_i^{\dagger }a_i$. Here, $S_i^{+} (S_i^{-})$ is the raising (lowering) “spin” operator and $a_i^{\dagger } (a_i)$ is a creation (annifiration) operator of the boson at the *i*th Tb site. We retain the quadratic terms of the boson operators since the higher order terms are considered to be irrelevant at least for the ground state.

**Figure 2:**
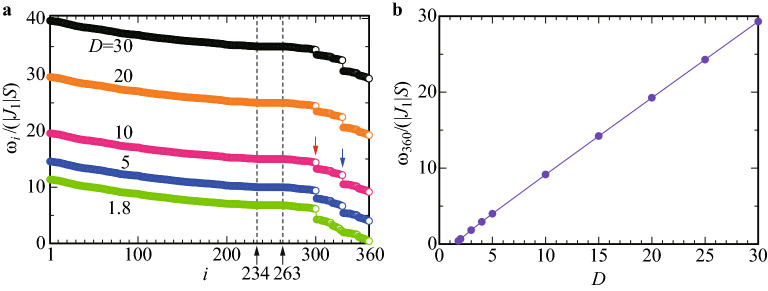
(**a**) $\omega _i$ vs *i* for $J_1=-1$ and $J_2=-1$ with $D=1.8, 5, 10, 20$, and 30. The dashed lines indicate $i=234$ and $i=263$. Red arrow points to $i=300$ and blue arrow points to $i=330$. (**b**) Magnetic anisotropy *D* dependence of the lowest excitation energy $\omega _{360}$.

By diagonalizing *H*, we obtain the energy spectrum $\omega _i$ for $J_1=-1$ and $J_2=-1$ as shown in Fig. 2a. For $D\ge 1.8$, positive $\omega _i$ for all *i* $(i=1,\ldots 360)$ is obtained (see Fig. 2b) reflecting the FM long-range order of the ferrimagnetic state for $\theta =80^{\circ }$ (see Fig. 1a,b) as the stable ground state. We find that there appear several gaps, among which the energy gaps are visible as the step structures at $i=300$ and $i=330$ (see red and blue arrows in Fig. 2a respectively). Interestingly, we find that $\omega _i$ from $i=234$ to 263 are degenerate, i.e., degeneracy is 30, irrespective of *D*. It turns out that these degenerated states give rise to unique magnetic excitation in the dynamical structure factor, which will be discussed later. Below we show the results for $D=10$ as a representative case for the QC Au–SM–Tb. For $D=10$, the lowest energy is $\omega _{360}/(|J_1|S)=9.15\equiv \Delta$ which is defined as the gap $\Delta$ and the largest energy is $\omega _{1}/(|J_1|S)=19.57$. The degenerated energy mentioned above is $\omega _i/(|J_1|S)=15.00$ for $i=234, \ldots 263$.

**Figure 3:**
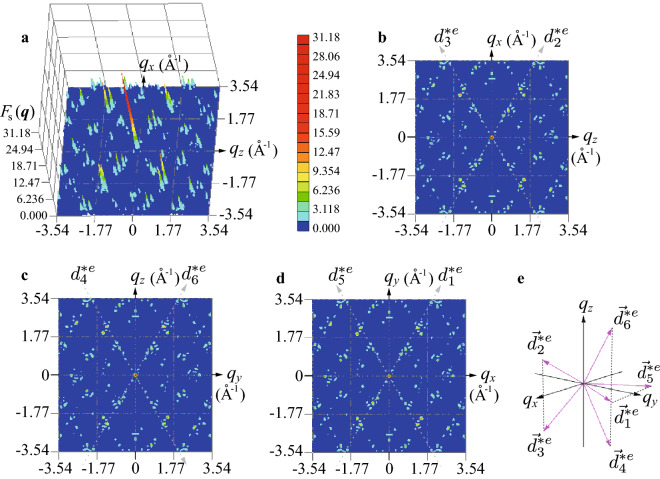
(**a**) Magnetic structure factor $F_s({{\varvec{q}}})$ in the $q_z$−$q_x$ plane for $q_y=0$. (**b**) Top view of (**a**). (**c**) Top view of $F_s({{\varvec{q}}})$ in the $q_y$−$q_z$ plane for $q_x=0$. (**d**) Top view of $F_s({{\varvec{q}}})$ in the $q_x$−$q_y$ plane for $q_z=0$. The gray dashed lines with arrows in (**b**), (**c**), and (**d**) denote the pseudo 5-fold axis defined in (**e**). (**e**) Primitive vectors in the six-dimensional reciprocal-lattice space ${\varvec d}_i^{*e}$ $(i=1,\ldots , 6)$ as the physical (external) space components.

### Static structure factor of magnetism

To clarify the magnetic structure in the reciprocal space, we calculate the magnetic structure factor

2 $$\begin{aligned} F_{s}({{\varvec{q}}})=\left\langle \left| \frac{1}{N}\sum _{i}{{\varvec{S}}}_{i}e^{i{{\varvec{q}}}\cdot {{\varvec{r}}}_i}\right| ^2 \right\rangle . \end{aligned}$$

The result of $$F_s({{\varvec{q}}})$$ in the $$q_z$$−$$q_x$$ plane for $$q_y=0$$ is shown in Fig. 3a. We find that a sharp peak appears at $${{\varvec{q}}}=\mathbf{0}$$. At finite $${{\varvec{q}}}$$, sharp peaks also appear with smaller intensities. The top view is shown in Fig. 3b, where the bright spots are located along the pseudo 5-fold axis indicated by the dashed line named the $$d_2^{e*}$$ line with an arrow. As shown in Fig. 3e, $$\mathbf {d}_i^{*e}$$ $$(i=1, \ldots , 6)$$ is the primitive vector of the six-dimensional reciprocal lattice space as the physical (external) space components^39^. The slope of the $$d_2^{e*}$$ line is 1.736 reflecting the real configuration of the Tb sites in the IC^12^ employed in this study and it is noted that the slope is to be $$\tau$$ in the case of the regular IC^39^. In Fig. 3b, the bright spots are also located along the dashed line with the negative slope − 1.736, which is named the $$d_3^{e*}$$ line. We also plot the top views of $$F_s({{\varvec{q}}})$$ in the $$q_y$$−$$q_z$$ plane for $$q_x=0$$ as Fig. 3c and in the $$q_x$$−$$q_y$$ plane for $$q_z=0$$ as Fig. 3d, where $$d_i^{e*}$$ lines $$(i=4,6$$ and $$i=1,5)$$ are drawn. From these results, we confirmed that the largest peak is located at $${{\varvec{q}}}=\mathbf{0}$$. This indicates that the uniform long-range order of the ferrimagnetic state on the IC (see Fig. 1a) is realized.

### Dynamical structure factor of magnetism

The dynamical magnetic structure factor is defined as $S_{\alpha \beta }({{\varvec{q}}},\omega )\equiv -\frac{1}{\pi }\mathrm{Im}G_{\alpha \beta }({{\varvec{q}}},\omega )$ $(\alpha , \beta =x, y, z)$^36^, where $G_{\alpha \beta }({{\varvec{q}}},\omega )=\frac{1}{N}\sum _{i,j}e^{i{{\varvec{q}}}\cdot ({{\varvec{r}}}_i-{{\varvec{r}}}_j)}G^{\alpha \beta }_{ij}(\omega )$ with

3 $$\begin{aligned} G^{\alpha \beta }_{ij}(\omega )=\left\langle \mathrm{GS}\left|S_{i\alpha }\frac{1}{\omega +E_0-H+i\eta }S_{j\beta }\right|\mathrm{GS}\right\rangle . \end{aligned}$$

Here, $|\mathrm{GS}\rangle$ is the ground state with $E_0$ being the ground-state energy and we set $\eta =10^{-6}$. We have calculated $S_{\alpha \alpha }({{\varvec{q}}},\omega )$ $(\alpha =x, y, z)$ and below we show the results of $S_{yy}({{\varvec{q}}},\omega )$ as a representative for the dynamical structure factor (see Supplementary Information for the results of $S_{xx}({{\varvec{q}}},\omega )$ and $S_{zz}({{\varvec{q}}},\omega )$).

**Figure 4:**
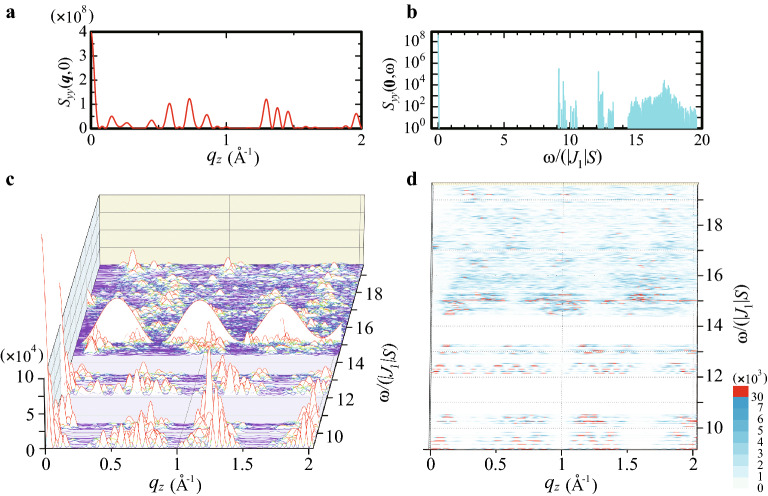
(**a**) Elastic component of the dynamical structure factor $S_{yy}({{\varvec{q}}},0)$ for ${{\varvec{q}}}$ along the $d_2^{e*}$ line. (**b**) The $\omega$ dependence of the dynamical structure factor $S_{yy}(\mathbf{0},\omega )$. (**c**) The dynamical structure factor $S_{yy}({{\varvec{q}}},\omega )$ for ${{\varvec{q}}}$ along the $d_2^{e*}$ line. (**d**) Top view of (**c**). (This figure is created by using Adobe Illustrator CS5 Version 15.1.0.).

The elastic component of the dynamical structure factor $S_{yy}({{\varvec{q}}},\omega =0)$ is shown for ${{\varvec{q}}}$ along the $d_2^{e*}$ line with $q_z\in [0,2]$ Å$^{-1}$ in Fig. 4a. The largest peak appears at ${{\varvec{q}}}={{\varvec{0}}}$ and several peak structures also appear for finite ${{\varvec{q}}}$, whose values are typically $O(10^8)$. As shown in Fig. 2a, in the magnetic excitation, the gap $\Delta$ is caused by the uniaxial anisotropy *D* arising from the CEF. To grasp the $\omega$ dependence of the dynamical structure factor, we plot $S_{yy}({{\varvec{q}}}=\mathbf{0},\omega )$ in Fig. 4b. For $\omega /(|J_1|S)\ge \Delta$, the spiky peak structure appears where the peak values are typically $O(10^4\sim 10^5)$.

Next, inelastic part of the dynamical structure factor $S_{yy}({{\varvec{q}}},\omega )$ above the CEF excitation gap is shown in Fig. 4c for ${{\varvec{q}}}$ along the $d_2^{e*}$ line with $q_z\in [0,2]$ Å$^{-1}$. The result shows the highly structured energy and wavenumber dependences. The largest peak appears at $({{\varvec{q}}}, \omega /(|J_1|S))=(\mathbf{0}, \Delta )$. The sharp peak structures appear in the lower energy regions above the CEF excitation gap: $\Delta \le \omega /(|J_1|S)<12.7$. Interestingly, it is remarkable that the successive mountain-like high intensity structure with a large periodicity $\Delta q\sim 0.6$ Å$^{-1}$ appears at $\omega /(|J_1|S)=15.0$. From the relation of wavenumber and wavelength $\Delta q=2\pi /\lambda$, the wavelength $\lambda$ is estimated to be $\lambda \sim 0.67a ~$Å. This roughly corresponds to the diameter of the IC $d=0.72a$ as shown in Fig. 1d. At $\omega /(|J_1|S)=15.0$, as $|{{\varvec{q}}}|$ increases, the intensity decreases with this periodicity. This mode is completely localized, which appears at the flat branch, i.e., non-dispersive constant $\omega$ with 30 degeneracy, reflecting the degenerated energy $\omega _i$ from $i=234$ to 263 shown in Fig. 2a. We confirmed that the degenerated localized modes appear when the N.N. interaction $J_1$ and N.N.N. interaction $J_2$ are equal irrespective of $\theta$ at least for $65^{\circ }\le \theta \le 80^{\circ }$ (see Supplementary Information). In Fig. 4d, above this localized mode, i.e. for $\omega /(|J_1|S)>15.0$, the small intensity structures appear in the broad range of the ${{\varvec{q}}}$-$\omega$ plane.

To analyze the wavenumber dependence of the magnetic excitation in more detail, we plot the top view of $S_{yy}({{\varvec{q}}},\omega )$ in Fig. 4d. We find that successive magnon-like mode i.e. sinusoidal-like shape with periodicity about $\Delta q\sim 0.15$ Å$^{-1}$ appears around $12.0<\omega /(|J_1|S)<12.7$. These sinusoidal-like modes further form the larger sinusoidal-like modes with $\Delta q\sim 0.3$  Å$^{-1}$ around $12.0<\omega /(|J_1|S)<13.3$. These series of excitations form unique hierarchical structure. The recursive structure i.e., self-similar structure was also reported in Fibonacci chain [36] and in two-dimensional octagonal tiling^37^. The similar self-similar structure can also be seen for the lower excitation energies $\Delta<\omega /(|J_1|S)<10.5$. We refer to these modes as pseudo-magnon modes hereafter. The pseudo magnon mode propagating along the 5-fold direction shown in Fig. 4d is inherent in the QC.

At $\omega /(|J_1|S)=15.0$, the flat branch appears, which is the localized mode as remarkably seen in Fig. 4c. Just below this branch, bright intensities exist and above the localized mode, the broad continuum-like structures appear for $15.0<\omega /(|J_1|S)<19.6$.

As shown in Fig. 4c,d, the high intensity appears in the low-energy region for $\omega /(|J_1|S)\le 15.0$ and in the high-energy region, the low intensity appears. This is characteristic of the magnetic excitation in the FM order of the ferrimagnetic state (see Fig. 1a,b) irrespective of the strength of the anisotropy. Namely, the similar feature was confirmed to appear for $1.8\le D\le 30.0$.

**Figure 5:**
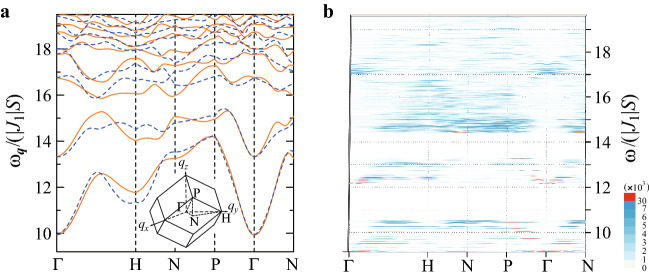
(**a**) Magnon dispersion is plotted as solid lines (dashed lines) in the 1/1 AC for ${{\varvec{q}}}$ $(-{{\varvec{q}}})$ along the symmetry line in the Brillouin zone of the body-center-cubic lattice shown in inset. (**b**) Dynamical structure factor $S_{yy}({{\varvec{q}}},\omega )$ in the QC for ${{\varvec{q}}}$ along the symmetry line of the inset.

### Comparison with 1/1 approximant crystal

To get insight into the emergence of the pseudo magnon mode in the QC, we calculate the magnon dispersion in the 1/1 AC. Namely, the model (1) is applied to the 1/1 AC (see Fig. 1c). On the atomic positions, here we employ the lattice structure of the 1/1 AC Au$_{70}$Si$_{17}$Tb$_{13}$^12^ with the lattice constant being $a=14.726~$Å. For the same parameter used in the QC, i.e., $J_1=-1$, $J_2=-1$, and $D=10$ with $S=6$, we confirmed that the FM long-range order of the ferrimagnetic state (Fig. 1b) is realized, as shown in Fig. 1c. Then, by applying the linear spin-wave theory^38^ to the model (1), we calculate the excitation energy of the magnon $\omega _{{\varvec{q}}}$ in the reciprocal space. The result for ${{\varvec{q}}}$ along the symmetry line in the bcc Brillouin zone is plotted as solid lines in Fig. 5a, where the number of the unit cell is taken as $64^3$. Similarly to the QC, owing to the uniaxial anisotropy $D=10$, the energy gap opens for $0<\omega _{{\varvec{q}}}/(|J_1|S)< 10$ in the magnon excitation. For $10\le \omega _{{\varvec{q}}}/(|J_1|S)\le 25.3$, the magnon excitation forms the dispersive energy bands. Here, for the comparison with the QC, the energy range of $\Delta \le \omega _{{\varvec{q}}}/(|J_1|S)\le 19.57$ is shown in Fig. 5a (for the result of the whole energy range, see Supplementary Information). It is noted that the length of the reciprocal lattice between the $\Gamma$ and H points is $2\pi /a=0.43~$Å$^{-1}$.

For comparison, we calculate $S_{yy}({{\varvec{q}}},\omega )$ in the QC for the same ${{\varvec{q}}}$ as that shown in Fig. 5a. The result for $\Delta \le \omega _{{\varvec{q}}}/(|J_1|S)\le 19.57$ is plotted as the top view in Fig. 5b. We see that the dispersive modes with the broad intensity start from the $\Gamma$ point at $\omega /(|J_1|S)=\Delta$ and also at $\omega /(|J_1|S)=12.5$, which seem to correspond to the dispersive magnon bands starting from the $\Gamma$ point at $\omega _\mathbf{q}/(|J_1|S)=10$ and 13.3 in Fig. 5a, respectively. These two magnon bands have the concave dispersions along the P-$\Gamma$-N line in Fig. 5a, which seem to correspond to the concave dispersive modes with the broad intensity along the P-$\Gamma$-N line around $\Delta \le \omega /(|J_1|S)\le 10.5$ and $12\le \omega /(|J_1|S)\le 13.3$ in Fig. 5b.

The quadratic dispersion $\omega =c{{\varvec{q}}}^2$ starting from the $\Gamma$ point at $\omega =\Delta$ is evaluated by using the high-intensity data (red data in Fig. 5b) in the QC. The magnon velocity ${{\varvec{v}}}=\partial \omega /(\partial {{\varvec{q}}})=2c{{\varvec{q}}}$ is about 4-times smaller than that estimated from the lowest-excitation-energy data in the vicinity of the $\Gamma$ point shown in Fig. 5a in the 1/1 AC. Furthermore, the convex magnon band at the H point around $\omega _{{\varvec{q}}}/(|J_1|S)=17.5$ in Fig. 5a seems to correspond to the mountain-like broad intensities at the H point around $\omega /(|J_1|S)=17$ in Fig. 5b. These results indicate that even in the QC, the magnetic excitation mode like magnon, i.e., the pseudo magnon mode, exists with broad width in their intensities.

### Non-reciprocal magnon in 1/1 approximant crystal

In the calculation of the magnon dispersion in Fig. 5a, we also plotted $\omega _{-{{\varvec{q}}}}$ as dashed lines. Notable is that there exists a remarkable difference between the solid line and the dashed line indicating $\omega _{{\varvec{q}}}\ne \omega _{-{{\varvec{q}}}}$. This implies that the non-reciprocal magnon appears in the present FM long-range order of the ferrimagnetic state. This is, to our best knowledge, the first discovery of the non-reciprocal magnon in the AC. Note that in the bcc lattice, the spatial inversion symmetry exists. The emergence of the non-reciprocal magnon is ascribed to the non-collinear “spin” configuration shown in Fig. 1b. To check this point, we calculated the magnon dispersion for the collinear “spin” configuration such as the FM long-range order where all the “spins” are aligned to the same direction in the 1/1 AC. In this case, $\omega _{{\varvec{q}}}=\omega _{-{{\varvec{q}}}}$ holds for all ${{\varvec{q}}}$, which indicates that the reciprocal magnon is realized (see Supplementary Information).

**Figure 6:**
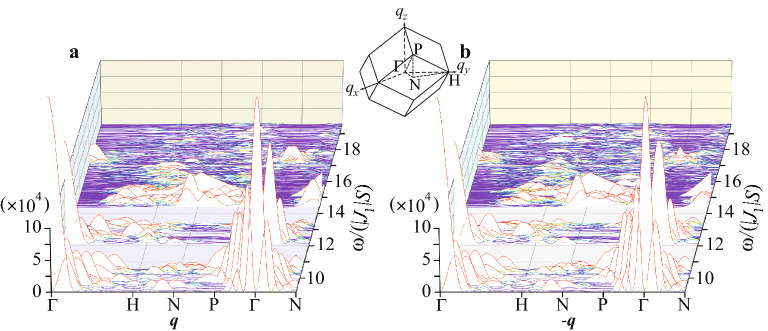
(**a**) Dynamical structure factor $S_{yy}({{\varvec{q}}},\omega )$ in the QC. **b** $S_{yy}(-{{\varvec{q}}},\omega )$ in the QC. In (**a**) and (**b**), ${{\varvec{q}}}$ is plotted along the symmetry line in the Brillouin zone of the body-center-cubic lattice of the 1/1 AC shown in inset. (This figure is created by using Adobe Illustrator CS5 Version 15.1.0.).

### Non-reciprocal magnetic excitation in quasicrystal

Next, we calculate the dynamical structure factor $S_{yy}({{\varvec{q}}},\omega )$ and also $S_{yy}(-{{\varvec{q}}},\omega )$ in the QC for ${{\varvec{q}}}$ along the symmetry lines in the bcc Brillouin zone of the 1/1 AC, as shown in Fig. 6a,b, respectively. By comparing both results, we find that there exist several differences between ${{\varvec{q}}}$ and $-{{\varvec{q}}}$, i.e., $S_{yy}({{\varvec{q}}},\omega )\ne S_{yy}(-{{\varvec{q}}},\omega )$. For instance, near the $\Gamma$ point around $\omega /(|J_1|S)\approx 17$, a clear difference exists. Indeed, the non-reciprocal magnon dispersion, i.e., deviation from the dashed line from the solid line, is shown to appear near the $\Gamma$ point around $\omega _{{\varvec{q}}}/(|J_1|S)\approx 17$ in Fig. 5a. Furthermore, around the *H* point for $17\le \omega /(|J_1|S)\le 19$, remarkable differences exist as seen in Fig. 6a,b. Actually, the $\omega _{{\varvec{q}}}\ne \omega _{-{{\varvec{q}}}}$ feature is prominent at the H point around $\omega _{{\varvec{q}}}/(|J_1|S)\approx 17$ in Fig. 5a. This is, to our best knowledge, the first discovery of the non-reciprocal magnetic excitation in the FM long-range order in the QC. This finding suggests that the non-reciprocal magnetic excitation appears generally in the icosahedral QC. This is because the alignment of the “spins” at the 12 vertices of the IC (see Fig. 1b) following the uniaxial anisotropy induced by the CEF at each rare-earth site inevitably becomes the non-collinear type.

### Discussion

We have clarified the static and dynamical structure factor in the FM long-range order of the ferrimagnetic state in the icosahedral QC. The static structure factor exhibits the largest peak at ${{\varvec{q}}}=\mathbf{0}$, which indicates the uniform long-range order of the ferrimagnetic state. By calculating the dynamical structure factor $S({{\varvec{q}}},\omega )$, the elastic part for $\omega =0$ is shown to have the largest peak at ${{\varvec{q}}}=\mathbf{0}$. The uniaxial anisotropy arising from the CEF causes the energy gap in the magnetic excitation so that the non-elastic part exhibits the intensity, whose order is 4 decades smaller than that of the elastic part, beyond the CEF gap. The inelastic $S({{\varvec{q}}},\omega )$ shows the highly structured energy and wavenumber dependences. In spite of no translational invariance in the QC, we have found the pseudo magnon mode with hierarchical structure propagating along the 5-fold direction inherent in the QC. We have also found the completely localized magnetic-excitation mode with periodicity characterized by the wavelength corresponding to the size of the IC in the wavenumber space as the degenerated modes.

This implies that this mode reflects the magnetic excitation on each icosahedron because of the cancellation of the dispersions of the excitation arising from symmetric interaction $J_1=J_2$ (see section II in Supplementary Information). The non-collinear and non-coplanar “spin” structure on the IC is shown to give rise to the non-reciprocal magnetic excitation in the QC as well as the non-reciprocal magnon in the AC. Our model and the results are expected to be relevant to the broad range of the rare-earth based icosahedral QCs and ACs with strong magnetic anisotropy. Hence, our findings as well as the method for the analysis developed in this study open a new research field of the magnetic dynamics in the QCs and ACs. It is noted that consideration of more detailed lattice structures of the Cd$_{5.7}$Yb-type QC and the examination of the effect of the system size i.e., boundary condition on magnetic dynamics are left for future studies.

## Methods

### Quasicrystal and approximant crystal

The rare-earth based QC and AC consists of the Tsai-type cluster with nested shell structures of polyhedrons. The rare-earth atom is located at the 12 vertices of the IC. The AC retains the periodicity as well as the common local atomic configuration to the QC. There exists a series of the ACs such as 1/1 AC, 2/1 AC, 3/2 AC, $\ldots$, where $F_n$ in the $F_{n+1}/F_{n}$ AC is the Fibonacci number $(F_{n+2}=F_{n+1}+F_n, F_1=F_2=1)$. In the 1/1 AC, the IC composed of the rare-earth atoms is distributed at the center and corner of the bcc lattice. As *n* increases, the size of the unit cell of the $F_{n+1}/F_{n}$ AC expands and for the $n\rightarrow \infty$ limit $(\lim _{n\rightarrow \infty }F_{n+1}/F_{n}=\tau )$ the size of the unit cell becomes infinite, which corresponds to the QC.

### Theory of magnetic excitation in quasicrystal

To calculate the magnetic excitation from the FM long-range order in the QC, we transform the spin operators in the model (1) into the boson operators. Since the ferrimagnetic state is a noncolinear magnetic state, it is convenient to introduce the local coordinate at each Tb site^40^. The unit vectors in the global *xyz* coordinate ${\hat{r}}_1={\hat{x}}$, ${\hat{r}}_2={\hat{y}}$, and ${\hat{r}}_3={\hat{z}}$ are expressed by the local orthogonal coordinate with the unit vector $\hat{{\varvec{e}}}^i_{3}$, whose direction is indicated by the polar angles $(\theta _i, \phi _i)$, as

4 $$\begin{aligned} \hat{{\varvec{r}}}_{\alpha }=R_{\alpha \beta }^{i}\hat{{\varvec{e}}}_{\beta }^{i} \end{aligned}$$

(see Fig. 1d). Here, $R^{i}$ is the rotation matrix defined as

5 $$\begin{aligned} R^{i}= \begin{bmatrix} \cos \theta _i\cos \phi _i &{} -\sin \phi _i &{} \sin \theta _i\cos \phi _i \\ \cos \theta _i\sin \phi _i &{} \cos \phi _i &{} \sin \theta _i\sin \phi _i \\ -\sin \theta _i &{} 0 &{} \cos \theta _i \end{bmatrix}. \end{aligned}$$

Then, the first term in Eq. (1) is expressed as

6 $$\begin{aligned} \sum _{\langle i,j\rangle }J_{i,j}({{\varvec{S}}}_i\cdot {{\varvec{e}}}_{\alpha }^i)({{\varvec{S}}}_j\cdot {{\varvec{e}}}_{\beta }^j)\sum _{\gamma }R_{\alpha ,\gamma }^{i}R_{\gamma ,\beta }^{j}. \end{aligned}$$

By using ${{\varvec{S}}}_i\cdot \hat{{\varvec{e}}}_1^i=(S_i^{+}+S_i^{-})/2$ and ${{\varvec{S}}}_i\cdot \hat{{\varvec{e}}}_2^i=(S_i^{+}-S_i^{-})/(2i)$ where $S_i^{+}$ and $S_i^{-}$ are raising and lowering “spin” operators, respectively, we apply the Holstein-Primakoff transformation^38^ to *H*. Namely, “spin” operators are expressed by the boson operators as $S_i^{+}=\sqrt{2S-n_i}a_i$, $S_i^{-}=a_i^{\dagger }\sqrt{2S-n_i}$ and ${{\varvec{S}}}_i\cdot \hat{{\varvec{e}}}_3^i=S-n_i$ with $n_i\equiv a_i^{\dagger }a_i$. We retain the quadratic terms with respect to $a_i^{\dagger }$ and $a_i$, which are considered to be at least valid for the ground state. In the noncollinear magnetic state as the hedgehog, anomalous terms such as $a_i^{\dagger }a_j^{\dagger }$ and $a_ia_j$ appear. The resultant *H* is expressed as

7 $$\begin{aligned} H=[\chi ^{\dagger } {\tilde{\chi }}]\Lambda \begin{bmatrix} \chi \\ {\tilde{\chi }}^{\dagger } \end{bmatrix}, \end{aligned}$$

where $\chi ^{\dagger }=(a^{\dagger }_1,a^{\dagger }_2,\ldots ,a^{\dagger }_N)$ and $\Lambda$ is the $2N\times 2N$ matrix. By performing the para unitary transformation

8 $$\begin{aligned} \begin{bmatrix} \zeta \\ {\tilde{\zeta }}^{\dagger } \end{bmatrix} ={{\mathcal {J}}} \begin{bmatrix} \chi \\ {\tilde{\chi }}^{\dagger } \end{bmatrix} \end{aligned}$$

where $\zeta =(\alpha _1, \alpha _2, \ldots , \alpha _N)$ and ${{\mathcal {J}}}$ is the para unitary matrix^41^, we obtain

9 $$\begin{aligned} H=[\zeta ^{\dagger } {\tilde{\zeta }}] \begin{bmatrix} {\bar{\omega }} &{} {\bar{0}} \\ {\bar{0}} &{} {\tilde{\omega }} \end{bmatrix} \begin{bmatrix} \zeta \\ {\tilde{\zeta }}^{\dagger } \end{bmatrix}. \end{aligned}$$

Here, ${\bar{\omega }}$ is the $N\times N$ diagonal matrix ${\bar{\omega }}=\mathrm{diag}(\omega _1, \omega _2, \ldots , \omega _N)$ with $\omega _i>0$, ${\tilde{\omega }}=\mathrm{diag}(\omega _N, \omega _{N-1}, \ldots , \omega _1)$, and ${\bar{0}}$ is the $N\times N$ matrix with all elements being zero.

## Supplementary Information

Supplementary Information.

## Data Availability

All the data supporting the findings are available from the corresponding author upon reasonable request.
