# Supplementary material for: Magnetic dynamics of ferromagnetic long range order in icosahedral quasicrystal
Source: Sci Rep. 2022 Jun 24;12:10792. doi: 10.1038/s41598-022-14796-5 (PMC9232551; doi:10.1038/s41598-022-14796-5)
Supplement: Supplementary file 1 — Supplementary Information. [file 41598_2022_14796_MOESM1_ESM.pdf]

Supplementary Information for
“ Magnetic dynamics of ferromagnetic long range order in icosahedral quasicrystal ”

Shinji Watanabe*

Department of Basic Sciences, Kyushu Institute of Technology,

Kitakyushu, Fukuoka 804-8550, Japan

(Dated: June 19, 2022)

I. Dynamical magnetic structure factors $S_{xx}(\boldsymbol{q},\omega)$ and $S_{zz}(\boldsymbol{q},\omega)$

In the main text, we have presented the results of the dynamical magnetic structure factor $S_{yy}(\boldsymbol{q},\omega)$. Here we show the results of $S_{xx}(\boldsymbol{q},\omega)$ and $S_{zz}(\boldsymbol{q},\omega)$ calculated on the basis of the model (1) in the main text for $J_1=-1$, $J_2=-1$, and $D=10$ with $S=6$ in the quasicrystal (QC). The results of $S_{xx}(\boldsymbol{q},\omega)$ and $S_{zz}(\boldsymbol{q},\omega)$ for $\boldsymbol{q}$ along the d_2^{e*} line (see Fig. 3e in the main text) are shown in Figs. S1 and S2, respectively.

As for the elastic part i.e., $S_{\alpha\alpha}(\boldsymbol{q},0)$ ($\alpha=x,z$), the largest peak appears at $\boldsymbol{q}=\boldsymbol{0}$ and several sharp peaks appear at finite $\boldsymbol{q}$ with the intensities of $O(10^8)$, as shown in Figs. S1a and S2a. Figure S1b shows the ω dependence of $S_{xx}(\boldsymbol{0},\omega)$. Due to the uniaxial anisotropy $D=10$ arising from the crystalline-electric-field (CEF), the energy gap $\Delta\equiv\omega_{360}/(|J_1|S)=9.15$ opens in the magnetic excitation. For $\omega/(|J_1|S)>\Delta$, the spiky peak structures appear with intensities of $O(10^4\sim10^5)$. The ω dependence of $S_{zz}(\boldsymbol{0},\omega)$ behaves similarly, as seen in Fig. S2b.

Figure S1c shows $S_{xx}(\boldsymbol{q},\omega)$ for $\omega/(|J_1|S)\geq\Delta$, which exhibits highly structured energy and wavenumber dependences. The top view is plotted in Fig. S1d. For $12<\omega/(|J_1|S)<12.8$, successive sinusoidal-like mode forms self-similar structure, giving rise to hierarchical structure. The similar self-similar structure also appears for $\Delta\leq\omega/(|J_1|S)<10.5$. These modes are pseudo-magnon modes in the QC as analyzed in the main text.

At $\omega/(|J_1|S)=15.0$, remarkable mountain-like structures with high intensity appear in Fig. S1c. This mode is completely localized, which appears at the flat blanch in the $\boldsymbol{q}$-ω plane as seen in Fig. S1d. This mode is energetically degenerate with 30 degeneracy, which is the consequence of the degenerated energy eigenvalues of ω_i for $i=234,\cdots,263$ in the model (1) (see Fig. 2a in the main text). Above this localized mode, the continuum-like small intensities appear in the broad $\boldsymbol{q}$-ω range, as shown in Fig. S1d.

Figure S2c shows $S_{zz}(\boldsymbol{q},\omega)$ for $\omega/(|J_1|S)\geq\Delta$, which also exhibits highly structured energy and wavenumber dependences. For $\Delta\leq\omega/(|J_1|S)<10.5$, the intensive peak structures appear, which form the hierarchical pseudo magnon modes as shown as the bright intensities in Fig. S2d. For $12<\omega/(|J_1|S)<12.8$, the successive sinusoidal-like modes with the self-similar structure also appear. At $\omega/(|J_1|S)=15.0$, three mountain-like structures with high intensity are remarkable in Fig. S2c, which appear at the completely flat blanch with 30 degeneracy, similarly to $S_{xx}(\boldsymbol{q},\omega)$ and $S_{yy}(\boldsymbol{q},\omega)$. Mainly above this localized mode,

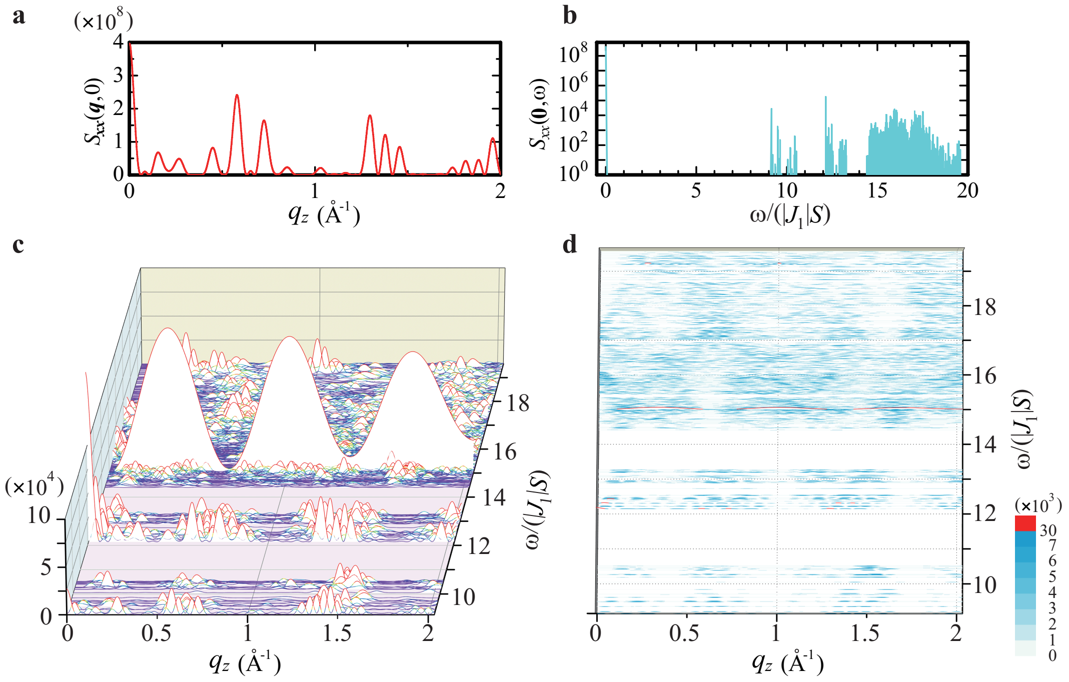

FIG. S1. (color online) **a** Elastic component of the dynamical structure factor $S_{xx}(\boldsymbol{q}, 0)$ for $\boldsymbol{q}$ along the d_2^{e*} line. **b** The ω dependence of the dynamical structure factor $S_{xx}(\mathbf{0}, \omega)$. **c** The dynamical structure factor $S_{xx}(\boldsymbol{q}, \omega)$ for $\boldsymbol{q}$ along the d_2^{e*} line. **d** Top view of **c**. (This figure is created by using Adobe Illustrator CS5 Version 15.1.0.).

the broad intensities continuously spread from each three mountain-like mode as shown in Fig. S2d.

II. Degenerated localized modes

In the main text, we have shown that the degenerated localized modes of magnetic excitation appear in the $\boldsymbol{q}$-ω plane of the QC in Figs. 4c and 4d. In this section, we discuss the parameter region for the emergence of the degenerated localized modes in the model (1) in the main text.

In the strong uniaxial-anisotropy limit of the model (1), the ground state of the QC has been determined in Ref. [1]. The FM long-range order of the ferrimagnetic state in the ground-state phase diagram is plotted as filled diamonds in Fig. S3a in the plane of J_2/J_1 and θ which is defined as the angle between the ordered-moment direction and the pseudo 5-fold axis (see Fig. 1b in the main text). Indeed, for the large anisotropy $D = 10$ in the model (1), we confirmed that all the energy eigenvalues ω_i are positive, i.e., $\omega_i > 0$ for

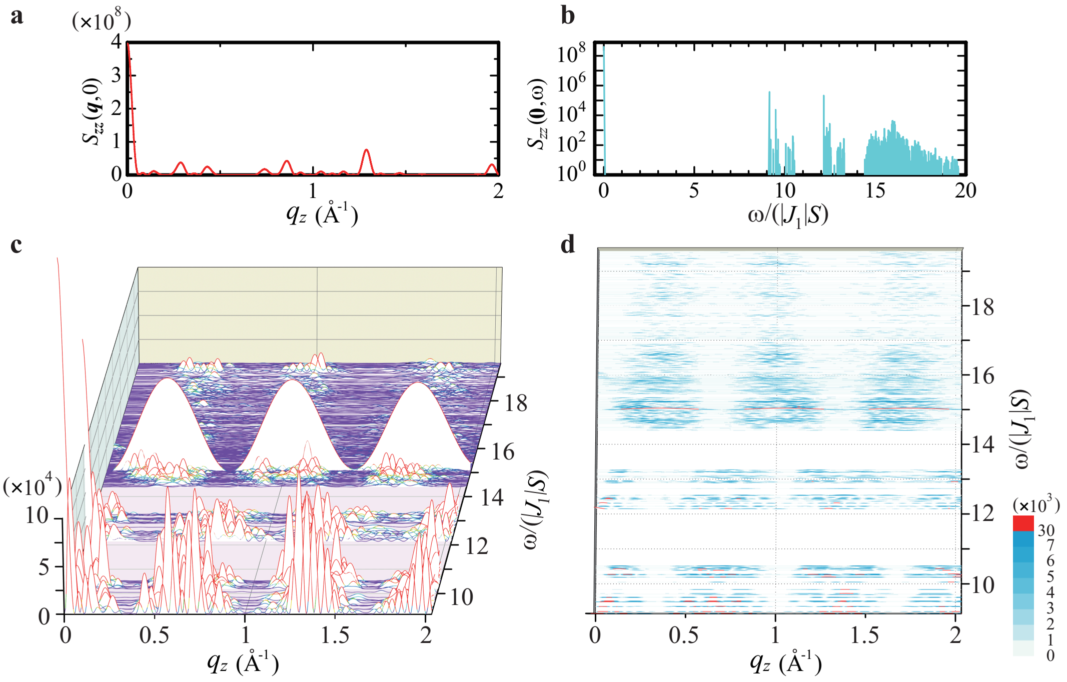

FIG. S2. (color online) **a** Elastic component of the dynamical structure factor $S_{zz}(\boldsymbol{q}, 0)$ for $\boldsymbol{q}$ along the d_2^{e*} line. **b** The ω dependence of the dynamical structure factor $S_{zz}(\boldsymbol{0}, \omega)$. **c** The dynamical structure factor $S_{zz}(\boldsymbol{q}, \omega)$ for $\boldsymbol{q}$ along the d_2^{e*} line. **d** Top view of **c**. (This figure is created by using Adobe Illustrator CS5 Version 15.1.0.).

$i = 1, \cdots, 360$, which confirms that the FM long-range order of the ferrimagnetic state is the stable ground state. Below we show the results for $D = 10$.

Figure S3b shows the energy ω_i for various J_2 with $J_1 = -1$ and $\theta = 80°$. For $J_2 = -1 = J_1$, the degenerated energy $\omega_i/(|J_1|S) = 15.0$ appears for $i = 234, \cdots, 263$ as presented in Fig. 2a in the main text. For $J_2 = -0.9$, the degeneracy disappears although almost flat ω_i is seen between the dashed lines in Fig. S3b. Reflecting the disappearance of the degeneracy at $\omega/(|J_1|S) = 15.0$, the mountain-like structures in $S_{yy}(\boldsymbol{q}, \omega)$ do not appear, as shown in Fig. S3c. We also confirmed that for $J_2 = -1.1$, the degeneracy of ω_i for $i = 234, \cdots, 263$ also disappears, giving rise to the disappearance of the mountain-like structures in $S_{yy}(\boldsymbol{q}, \omega)$, as shown in Fig. S3d. These results indicate that the degenerated localized modes appear when $J_2 = J_1$ or at least in the very vicinity of $J_2 = J_1$.

We confirmed that this also holds for the different θ. Figure S4a shows the energy ω_i for various J_2 with $J_1 = -1$ and $\theta = 65°$. For $J_2 = -1 = J_1$, the degenerated energy $\omega_i/(|J_1|S) = 15.0$ appears for $i = 235, \cdots, 264$. At $\omega/(|J_1S|) = 15.0$, the mountain-like

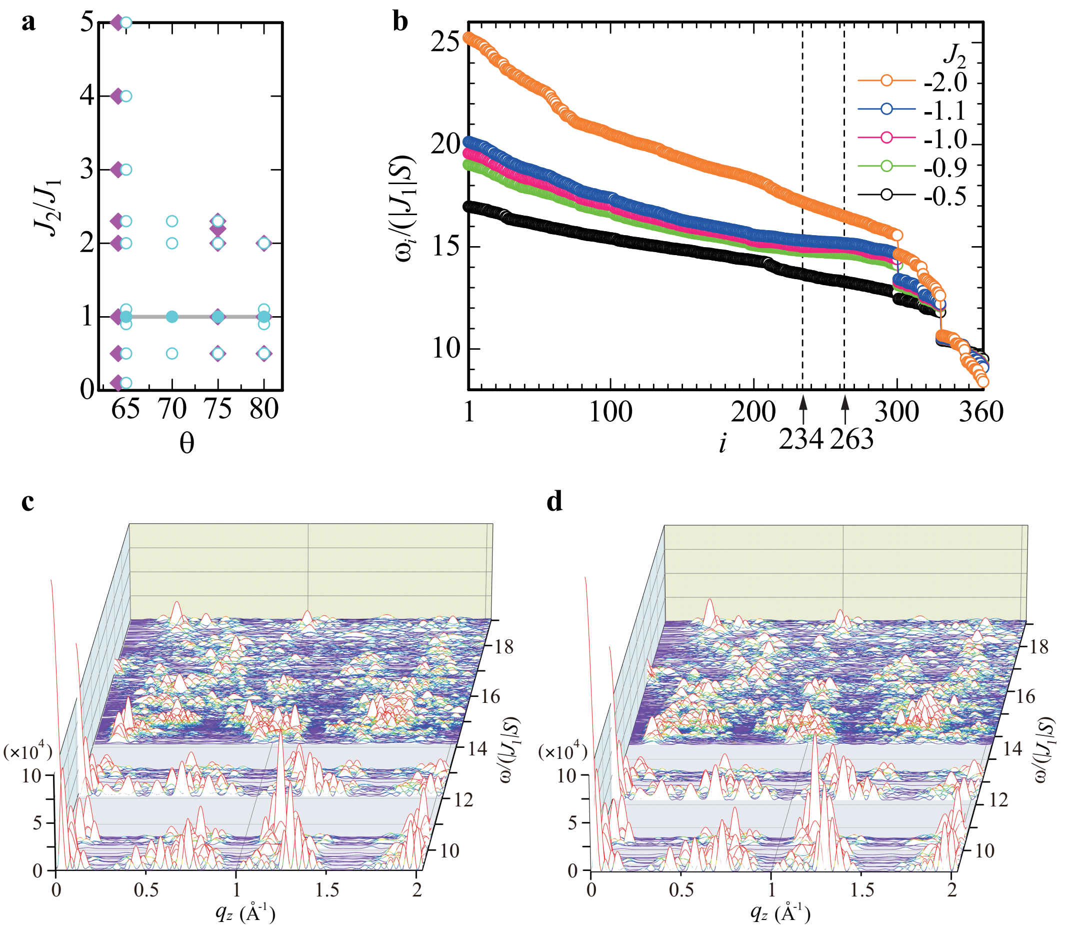

FIG. S3. (color online) **a** FM long-range order of the ferrimagnetic state in the QC realized in the θ-J_2/J_1 phase diagram determined by the magnetic model corresponding to the strong anisotropy limit of the model (1) (filled diamond) [1] and by the model (1) for $D = 10$ (open circle). The degenerated localized modes appear at the filled circles. The gray line at $J_2 = J_1$ is the guide for the eyes. **b** ω_i vs i for various J_2 with $J_1 = -1$ and $D = 10$. $S_{yy}(\boldsymbol{q}, \omega)$ for $\boldsymbol{q}$ along the d_2^{e*} line for **c** $J_2 = -0.9$ and **d** $J_2 = -1.1$ at $J_1 = -1$ and $D = 10$ with $\theta = 80°$. (This figure is created by using Adobe Illustrator CS5 Version 15.1.0.).

structures with high intensity appears in $S_{yy}(\boldsymbol{q}, \omega)$ as shown in Fig. S4b. In the ground-state phase diagram Fig. S3a, we plot the filled circles at which the degenerated energies appear for $J_2 = -1 = J_1$ and the gray line as the guide for the eyes.

III. Magnon excitation in the ferromagnetic long-range order of the ferrimagnetic state in 1/1 approximant crystal

In this section, we present the full energy bands of the magnon excitation in the 1/1

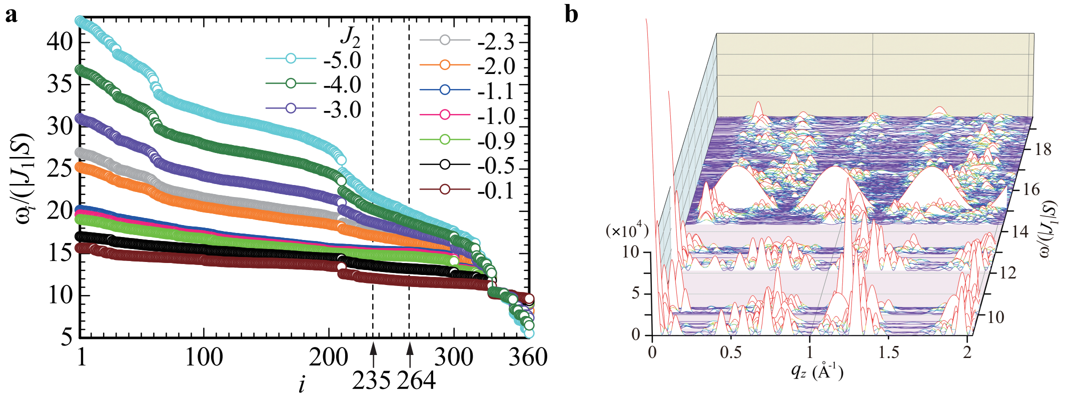

FIG. S4. (color online) **a** ω_i vs i for various J_2 at $J_1 = -1$, $D = 10$ and $\theta = 65^\circ$ with $S = 6$ in the QC. **b** $S_{yy}(\boldsymbol{q}, \omega)$ for $\boldsymbol{q}$ along the d_2^{e*} line for $J_1 = J_2 = -1$ at $D = 10$ with $\theta = 65^\circ$ in the QC. (This figure is created by using Adobe Illustrator CS5 Version 15.1.0.).

approximant crystal (AC). We apply the model (1) with $S = 6$ in the main text to the 1/1 AC. Here we employ the lattice structure of 1/1 AC $Au_{70}Si_{17}Tb_{13}$ with the lattice constant being $a = 14.726$ Å [2]. For $J_1 = -1$, $J_2 = -1$, and $D = 10$, we have confirmed that the ferromagnetic (FM) long-range order of the ferrimagnetic state is realized, as shown in Fig. S5a. On the basis of the linear spin-wave theory [3], we calculate the wavenumber dependence of the energy of the magnon excitation $\omega_{\boldsymbol{q}}$. Figure S5b shows $\omega_{\boldsymbol{q}}$ for $\boldsymbol{q}$ (solid lines) along the symmetry lines in the Brillouin zone of the body-center-cubic (bcc) lattice shown in Fig. S5c. In the unit cell of the bcc lattice (see Fig. S5a), there exist 24 Tb sites. Hence, there appear 24 energy bands of magnon. In the numerical calculation, the number of the unit cell is taken as 64^3 under the periodic boundary condition. Owing to the uniaxial anisotropy $D = 10$ arising from the CEF, the lowest energy of the magnon excitation is finite, i.e., the energy gap opens. Beyond the gap, i.e., $\omega_{\boldsymbol{q}}/(|J_1|S) > 10$, the dispersive energy bands appear.

In Fig. S5b, we also plot $\omega_{-\boldsymbol{q}}$ (dashed lines) for $\boldsymbol{q}$ along the symmetry lines in the Brillouin zone of the bcc lattice. The results show that there exist remarkable differences between the solid line and the dashed line, i.e., $\omega_{\boldsymbol{q}} \neq \omega_{-\boldsymbol{q}}$. This indicates that the non-reciprocal magnon appears in the 1/1 AC.

IV. Magnon excitation in the collinear FM long-range order in 1/1 approximant crystal

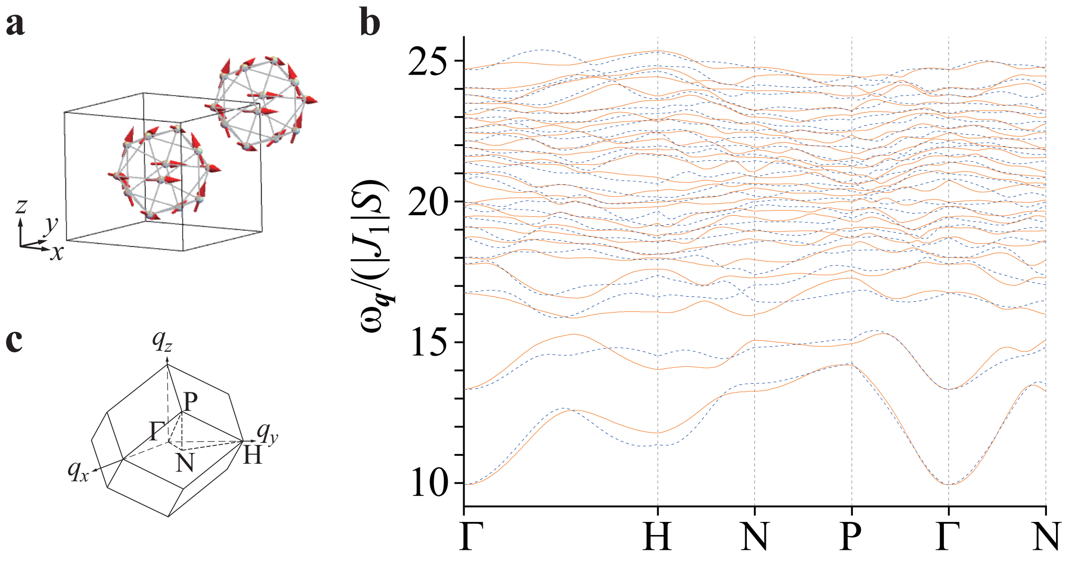

FIG. S5. (color online) **a** FM long-range order of the ferrimagnetic state on the IC in the 1/1 AC. The framed box is the unit cell of the bcc lattice. **b** Magnon energy bands $\omega_{\boldsymbol{q}}$ (solid lines) and $\omega_{-\boldsymbol{q}}$ (dashed lines) for $\boldsymbol{q}$ along the symmetry lines in the 1/1 AC for $J_1 = -1$, $J_2 = -1$, and $D = 10$ with $S = 6$. **c** The Brillouin zone of the bcc lattice. (This figure is created by using Adobe Illustrator CS5 Version 15.1.0.).

To get insight into the origin of the emergence of the non-reciprocal magnon, for comparison, we also calculate the magnon dispersion in the collinear FM long-range order in the 1/1 AC. We consider the ground state where all "spins" are aligned to the z direction on the IC as shown in Fig. S6a forming the FM long-range order in the 1/1 AC as Fig. S6b. Although in reality, this state is unlikely to be realized under the CEF at each Tb site, here we assume this state as the ground state. By applying the model (1) with $S = 6$ in the main text to the 1/1 AC, we calculate the wavenumber dependence of the energy of the magnon excitation $\omega_{\boldsymbol{q}}$ by the linear spin-wave theory [3] for $J_1 = -1$, $J_2 = 0$, and $D = 10$. The result of $\omega_{\boldsymbol{q}}$ for $\boldsymbol{q}$ along the symmetry lines in the Brillouin zone of the bcc lattice is shown as solid lines in Fig. S6c. The magnon excitation beyond the energy gap due to the CEF uniaxial anisotropy exhibits the dispersive energy bands.

We also plot $\omega_{-\boldsymbol{q}}$ as dashed lines in Fig. S6c. The results show that there is no difference between the solid line and dashed line, which indicates $\omega_{\boldsymbol{q}} = \omega_{-\boldsymbol{q}}$. Namely, reciprocal magnon appears in the present collinear FM long-range order, which is in sharp contrast to the non-collinear and non-coplanar "spin" alignment of the ferrimagnetic state in Sec. III.

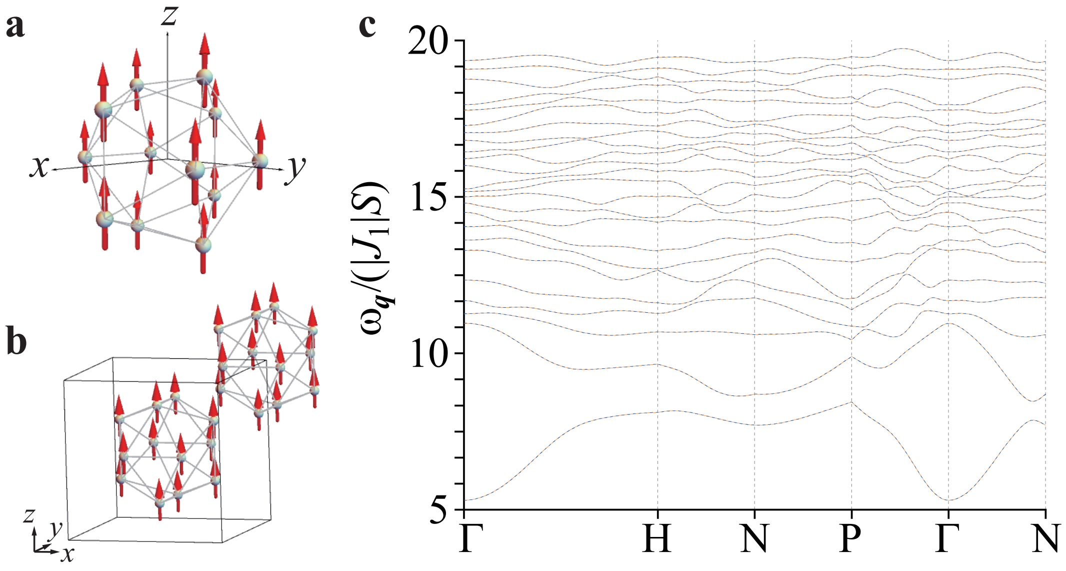

FIG. S6. (color online) **a** Collinear FM state on the IC. **b** FM long-range order of the collinear FM state in the 1/1 AC. The framed box is the unit cell of the bcc lattice. **c** Magnon energy bands $\omega_{\boldsymbol{q}}$ (solid lines) and $\omega_{-\boldsymbol{q}}$ (dashed lines) for $\boldsymbol{q}$ along the symmetry lines in the 1/1 AC for $J_1 = -1$, $J_2 = 0$, and $D = 10$ with $S = 6$. (This figure is created by using Adobe Illustrator CS5 Version 15.1.0.).

[1] Watanabe, S. Topological magnetic textures and long-range orders in terbium-based quasicrystal and approximant, *Proc. Natl. Acad. Sci. USA.* **118 (43)**, e2112202118 (2021).

[2] Hiroto, T. *et al.* Noncoplanar ferrimagnetism and local crystalline-electric-field anisotropy in the quasicrystal approximant $Au_{70}Si_{17}Tb_{13}$, *J. Phys.: Condens. Matter* **32**, 415802 (2020).

[3] Holstein, T. & Primakoff, H. Field dependence of the intrinsic domain magnetization of a ferromagnet, *Phys. Rev.* **58**, 1098 (1940).
